# A practical guide for researchers and reviewers using the ABCD Study and other large longitudinal datasets

**DOI:** 10.1016/j.dcn.2022.101115

**Published:** 2022-05-20

**Authors:** Natalie M. Saragosa-Harris, Natasha Chaku, Niamh MacSweeney, Victoria Guazzelli Williamson, Maximilian Scheuplein, Brandee Feola, Carlos Cardenas-Iniguez, Ece Demir-Lira, Elizabeth A. McNeilly, Landry Goodgame Huffman, Lucy Whitmore, Kalina J. Michalska, Katherine SF Damme, Divyangana Rakesh, Kathryn L. Mills

**Affiliations:** a Department of Psychology, University of California Los Angeles, Los Angeles, CA, USA; b Department of Psychology, University of Michigan, Ann Arbor, USA; c Division of Psychiatry, Centre for Clinical Brain Sciences, University of Edinburgh, UK; d Department of Psychology, University of Oregon, USA; e Institute of Education and Child Studies, Leiden University, The Netherlands; f Department of Psychiatry and Behavioral Sciences, Vanderbilt University Medical Center, Nashville, TN, USA; g Department of Population and Public Health Sciences, University of Southern California, Los Angeles, CA, USA; h Department of Psychological and Brain Sciences, University of Iowa, IA, USA; i Department of Neuroscience, University of Georgia, GA, USA; j Department of Psychology, University of California Riverside, Riverside, CA, USA; k Institute of Developmental Science, Northwestern University, Chicago, IL, USA; l Melbourne Neuropsychiatry Centre, Department of Psychiatry, University of Melbourne and Melbourne Health, Victoria, Australia; m PROMENTA Research Center, Department of Psychology, University of Oslo, Norway

**Keywords:** Adolescent Brain Cognitive Development (ABCD) Study, Adolescent development, Longitudinal research, Open research, Practical guide

## Abstract

As the largest longitudinal study of adolescent brain development and behavior to date, the Adolescent Brain Cognitive Development (ABCD) Study® has provided immense opportunities for researchers across disciplines since its first data release in 2018. The size and scope of the study also present a number of hurdles, which range from becoming familiar with the study design and data structure to employing rigorous and reproducible analyses. The current paper is intended as a guide for researchers and reviewers working with ABCD data, highlighting the features of the data (and the strengths and limitations therein) as well as relevant analytical and methodological considerations. Additionally, we explore justice, equity, diversity, and inclusion efforts as they pertain to the ABCD Study and other large-scale datasets. In doing so, we hope to increase both accessibility of the ABCD Study and transparency within the field of developmental cognitive neuroscience.

## 1 Introduction

The recent increase in large, widely available longitudinal datasets presents unprecedented opportunities for researchers to investigate developmental phenomena across myriad domains. Working with these datasets warrants careful attention to the complexities involved in study planning, design, analysis, and dissemination. The primary purpose of this guide is to provide a scaffolding of related considerations for researchers working with data from the Adolescent Brain Cognitive Development (ABCD) Study®, with the hope that these considerations can also be applied to other large, longitudinal datasets in the field of developmental cognitive neuroscience. The ABCD Study consists of ~11,800 youth, aged 9–10 years at baseline, who were recruited from 21 sites across the US with the aim of creating a population-level, socio-demographically-diverse sample (Garavan et al., 2018). The considerations highlighted within this paper take into account both the strengths and constraints of secondary data analysis, so as to prepare researchers at the outset of their projects. The following considerations were selected with the goal of making a valuable resource to the field—the ABCD Study—more accessible, while also increasing transparency and reproducibility within developmental cognitive neuroscience research more broadly. We first describe study design features of the ABCD Study, and then provide detailed step-by-step considerations that researchers should consider when preparing to work with ABCD data. We also highlight considerations of justice, equity, diversity, and inclusion efforts as they relate to the ABCD Study and other large-scale studies in the population. Our hope is that this paper provides a relevant guidebook for researchers embarking on projects using the ABCD dataset as well as for reviewers of studies using the ABCD dataset. As such, this guide is not comprehensive in its scope, and refers to relevant publications and resources for more in-depth information on the topics covered.

## 2 Features of the ABCD Study

In this section, we introduce broad characteristics and design features of the ABCD Study that researchers should keep in mind when working with ABCD data. Many of these characteristics are also applicable to other large-scale longitudinal studies.

The initial goal of the ABCD Study was to examine risk and resilience factors associated with the development of substance use disorders (i.e., cannabis), but the aims of the project have since expanded and now will inform population-level inferences about the biopsychosocial correlates of mental and physical health in the second decade of life (Barch et al., 2021). As with any longitudinal study, understanding the purpose of the study, how study data were collected, and any historical cohort effects on data collection is essential. The following section will delineate key features of ABCD Study including the unique study design, and potential cohort effects that may affect data collection and analysis.

### 2.1 ABCD is a developmental study

The ABCD Study represents a tremendous effort to capture developmental processes from late childhood into adolescence. One key goal of the ABCD study is to capture the dynamic and ongoing changes that occur during multiple developmental periods, and researchers have a responsibility to account for the developmental goals and processes that occur in the given age range assessed in a given study. However, many factors influence development and, most likely, researchers will not be able to account for all these factors in one study design (Nketia et al., 2021, Simmons et al., 2021). It is therefore important to think about how research questions are theorizing, assessing, and modeling the intended developmental change (King et al., 2018). As we detail further in this guide, understanding the study design and assessment schedule of the ABCD Study is crucial for considering how research question(s) address development. One of the many strengths of the ABCD Study is that its large sample and longitudinal design allow for investigations measuring *individual differences* in developmental processes. We therefore encourage researchers interested in making use of the longitudinal nature of the data to consult the ABCD website (https://abcdstudy.org/scientists/) which includes information about the study design, protocols, data sharing, and work groups. Other openly-available resources (e.g., https://abcdworkshop.github.io, https://www.abcd-repronim.org/about.html) have also been created and are maintained by a broad community of researchers.

### 2.2 ABCD is a cohort design

The ABCD Study is a longitudinal cohort study following youth and their families from pre-adolescence to young adulthood with annual lab-based assessments and bi-annual imaging acquisitions. Cohort designs have several advantages over other study designs, primarily that they can be used to determine the incidence and etiology of developmental (or pathological) processes while minimizing recall bias and maximizing statistical power for detecting population-level effects (Morrow, 2010). However, cohort designs also require a substantial time and financial commitment and may be slower to reveal developmental effects (particularly as compared to an accelerated cohort design; Garavan et al., 2018). This may lead to attrition issues, undue burden on participants, and compromises between the depth and breadth of measures used and the data collected. For instance, many brief or shortened measures have been administered in ABCD and some are only administered every other year (such as the NIH Toolbox). Further, although saliva samples and other biological data have been collected, their use may be limited due to collection issues inherent in large sample designs (e.g., getting participants to donate saliva around the same time of day; see Cheng et al., 2021; Uban et al., 2018 for further information).

In addition to these concerns, cohort effects can be a threat to external validity (Morrow, 2010). As with any longitudinal study, historical events can disrupt time-sensitive data collection (e.g., yearly collection of specimens and brain scans), but also provide unprecedented opportunities for novel research questions and hypothesis generation. For instance, the novel coronavirus pandemic (COVID-19) that emerged in late 2019 created challenges for data collection. Although the first wave of data collection was completed in 2018, the second wave of scans was suspended in mid-March 2020 due to pandemic-related stay-at-home orders. Data collection was adapted to include virtual visits and incorporated additional surveys on healthcare access, COVID-19 symptoms, and attitudes towards vaccination that may be helpful for researchers interested in investigating the lasting impact of COVID-19 on mental and physical health in adolescents.

In-person scans resumed in August 2020, but as of March 2022, the majority of the assessment data is still being collected remotely due to safety concerns. Thus, researchers should be aware that data collected from 2020 to 2022 may have been collected differently than in previous sessions and will be collected differently in the future. This could have both positive and negative consequences: Some participants may have benefited from completing assessments virtually because they did not need to arrange transportation or find time to travel to research sites, but others may not have had access to quiet or safe environments to complete testing. Although attrition data due to the pandemic are not yet available, researchers should be aware that individuals were differentially affected by pandemic-related stressors and that racial/ethnic minorities and individuals from low-income backgrounds may have faced additional challenges during the pandemic affecting their ability to participate in the second wave of ABCD data collection. For example, research using the ABCD dataset suggests that non-White and/or Spanish-speaking families experienced more financial worry and increased food insecurity during the COVID-19 pandemic (Yip et al., 2022). This could translate to reduced rates of study participation, particularly if there are resource issues (e.g., internet connectivity, availability of personal electronic devices) or increased relocation among racial/ethnic minorities and individuals from low-income backgrounds due to parental loss or changes in employment status. Moreover, a study analyzing participant retention in the ABCD Study found that, prior to the COVID-19 pandemic, parental education level and employment status were the most consistent indicators of risk for missed visits and study withdrawal (Feldstein Ewing et al., 2022); these challenges were likely exacerbated during the pandemic.

Cohort effects can also have implications for the interpretation and generalizability of findings. That is, it can be difficult to disentangle age-related effects from historical effects that take place during the same developmental window. This has important implications for researchers working with the ABCD Study, which broadly aims to understand trajectories of development and individual risk for psychopathology at the population level. For example, recent longitudinal work from 12 samples of youth ages 9–18 in the Netherlands, Peru, and the United States reported significant increases in depressive symptoms during the first six months of the COVID-19 pandemic (Barendse et al., 2021, Racine et al., 2021). Increases in internalizing symptoms during COVID-19 pandemic have been reported in other adolescent studies as well (Crescentini et al., 2020, Guazzelli Williamson et al., 2021). This poses a challenge for researchers who are interested in pursuing questions relating to development and mental health but plan to use data collected during the pandemic. It is therefore important to incorporate potentially confounding measures into statistical models when possible and to address these limitations when interpreting results. Some of these variables (e.g., detailed youth-and parent-report data on changes in routines, mental health, and experiences of racism during the pandemic) are already available in the ABCD dataset and others may become available as data collection continues (see the ABCD January 2021 Newsletter for further information).

As the ABCD Study continues, researchers will need to be aware of several other potential cohort effects that may bias the data, including practice effects. For instance, although COVID-19 represents perhaps the most significant disruption to adolescents’ daily lives, this period has also seen political upheaval and social movements (e.g., Black Lives Matter) come to the forefront of everyday life. Understanding the contextual effects of these events on data collection in ABCD is essential for conducting nuanced and culturally relevant research. The ABCD research team has already collected and released supplemental data related to the COVID-19 pandemic (ABCD COVID Rapid Response Research Survey; ABCD Data Release 3.0), natural disaster exposures (e.g., Hurricane Irma; Hoffman et al., 2019a, Hoffman et al., 2019b), and racial justice protests (Baskin-Sommers et al., 2021). Researchers can also use residential or census data if they are interested in investigating other cohort events that may affect a subset of their participants (see Chieh Fan et al., 2021 for geocoding protocols). As the ABCD Study is constantly evolving, researchers should subscribe to the ABCD newsletter (https://abcdstudy.org/newsletters/) and check release notes (which can be found on the NDA website in the ABCD curated annual release summary pages) to get up-to-date information about new initiatives or rapid data releases. Researchers may also be able to contact working group members (https://abcdstudy.org/scientists/workgroups/) for further information about ongoing substudies.

### 2.3 ABCD data are nested

The ABCD Study is unique in that it is oversampled for siblings and twins. This is important for understanding the contributions of genetics, shared family environments, and non-shared environments to developmental processes that contribute to individual differences (see Iacono et al., 2018 for further information and modeling specifics). However, researchers interested in using ABCD data, but uninterested in twin- or sibling-effects, then face an important question: How to best model the nested structure of the data? When nested data is used and statistical dependencies are not modeled appropriately, standard errors are inflated, potentially leading to inaccurate statistical conclusions and higher rates of Type I error (Grimm et al., 2016). There are many methods available that will account for the nested structure of ABCD data. Among these, multilevel models, hierarchical linear models, and Bayesian models have become increasingly popular. The mathematical bases and assumptions of these models have been established (Etz and Vandekerckhove, 2018, Raudenbush and Bryk, 2002, Sterba, 2014) and detailed primers and statistical packages are available elsewhere (see Heeringa & Berglund, 2020 for ABCD-relevant guidelines). Here, we provide several overarching questions and considerations:

**Table:** 

• As siblings and twins are nested within families and families are nested within-site, three-level models with random effects for family and site should be considered. • If using a hold-out approach, consider how twins or siblings are being split across subsamples. • As longitudinal data continues to be released, nesting time within individuals (within families and sites) could be considered and used if well-powered. • For certain types of physiological data, additional nesting may be necessary. For example, it may be important to consider batch effects for hormone data or scanner effects for MRI data (as three different scanners were used in ABCD data collection).

### 2.4 ABCD data can and should be used for hypothesis generation

The ABCD Study provides a great framework for studying development with a large sample of children and families across the United States. Although the ABCD Study includes many important factors and aspects of development, it is not all-encompassing. Given the large range of interests in the ever-growing field of developmental science, there will be measures not included or captured in the ABCD Study. While the ABCD Study is one of the largest and best equipped studies to broadly investigate adolescent development to date, no single study can capture the full dynamic landscape of development; thus, ABCD data might be best used for achieving hypothesis generation. These novel hypotheses generated by the ABCD Study may be better suited for researchers to experimentally test or more intensely study in their own research labs. Therefore, to continue moving the field forward, researchers should aim to create a balance between using the ABCD Study to learn about development and conducting independent studies designed to address the specific hypothesis generated from the ABCD Study.

## 3 Think before you do: necessary steps and considerations when preparing to work with ABCD data

In this section, we outline a series of recommendations for researchers to consider before and while working with ABCD data. It is hoped that adopting as many of these considerations as possible will aid the reproducibility and transparency of results emerging from the ABCD Study.

### 3.1 Determining whether ABCD data are well-suited to address your research question

When considering starting a project using ABCD data, it is important to first gain an understanding of what questions can be answered from the study. This step will involve reviewing the study’s overall design, methods for data collection, measures included at each wave (Barch et al., 2018, Casey et al., 2018, Hagler et al., 2019) and what, if any, changes are expected to be made in future waves of the study (for an example, see Barch et al., 2021). Reviewing preregistered studies that plan to use ABCD data (https://osf.io/search/) and existing studies of ABCD data found on the ABCD Study website (https://abcdstudy.org/publications/), can aid in conceptualizing what types of questions can be answered using ABCD data. A central consideration is whether the ABCD dataset is appropriate to answer your research question, for example, in terms of the age range, developmental stage, or sampling design.

Additionally, the National Institute of Mental Health Data Archive (NDA) requires researchers to create an NDA Study, which generates an associated DOI, prior to publishing a manuscript that uses NDA-based data, which includes the ABCD dataset. Existing NDA studies can be found on the “Data from papers” page (https://nda.nih.gov/general-query.html?q=query=studies%20~and~%20orderBy=id%20~and~%20orderDirection=Ascending) on the NDA website, which provides comprehensive information related to the portion of the sample used, the measures involved, and the type of data analyses conducted and is accessible to all researchers, without needing NDA approval. It is important to note that due to the broad accessibility of ABCD data, it is possible that a similar research question to your own is already being asked of the ABCD dataset. However, having multiple research groups tackle the same question is a useful exercise to test the robustness of both our methods and results as well as aiding collaborative work.

As research questions are being formulated, it is important to next consider the tools available that enhance transparency and reproducibility in developmental research, especially in regards to secondary data analysis (Kievit et al., 2021, Weston et al., 2019). Preregistrations and registered reports are two approaches to transparently report hypotheses (whether exploratory or confirmatory) and identify variables of interest, inclusion/exclusion criteria, statistical approaches, and analytical decisions (Nosek et al., 2018). The importance of using open science practices during these preliminary steps of one’s study, prior to the data analysis phase, has been emphasized within the field of developmental cognitive neuroscience (see Flournoy et al., 2020 and Klapwijk et al., 2021 for further information on this topic). Informed by this guidance, the following considerations describe additional factors to be incorporated within an ABCD-based study’s preregistration or registered report that will ultimately enhance the credibility of its findings, further contributing to our understanding of adolescent development.

### 3.2 Gaining access to ABCD data

Completing the required Data User Certificate for access to the ABCD data can be undertaken alongside the considerations outlined in the previous section so that you start planning your project while waiting for access to the data. Direct access to ABCD data requires approval from the NDA Data Access Committee (see nda.nih.gov/abcd/request-access for full instructions). In brief, researchers must be associated with a research institution that has an NIH-recognised Signing Official. The Signing Official at the researcher’s institution must review and sign the DUC, agreeing to comply with the NDA data use terms and conditions, before the DUC is reviewed by the NDA Data Access Committee. Obtaining institutional signoff can take a considerable amount of time so we encourage researchers to plan accordingly. Once the DUC is approved, researchers with NDA access are also granted access to the Data Exploration Analysis Portal (DEAP; deap.nimhda.org), a statistical analysis platform wherein researchers can readily engage with the ABCD data, such as exploring variables, downloading data, or running analyses.

### 3.3 Choosing your variables

Even before data access is granted, researchers should examine the ABCD data dictionary, which is publicly available on the NDA website (nda.nih.gov/data_dictionary.html), to identify variables of interest. The ABCD Study includes a wide array of data on youths’ and families’ environmental context, mental and physical health, behavior, genes, and neurocognitive development. With this extensive number of measures comes a myriad of choices necessary to decide which are relevant and necessary to your specific study. Choosing and working with the specific ABCD data of interest for your study requires an understanding of the following: 1) downloading ABCD data, 2) the structure/organization of the data files, 3) the availability of the data, 4) covarying with caution, and 5) the details of the data, each of which are detailed in the following sections.

### 3.4 Downloading ABCD data

Once a researcher has NDA-approved access to ABCD data, the data can be downloaded from the curated data releases housed on the NIMH Data Archive in the form of.txt or.Rds files. DEAP can also be used to explore and analyze the ABCD data as well as used to curate data frames with your variables of interest, which can then be downloaded as.txt or.Rds files. This latter function can be of particular use if you need to create a dataframe that combines measures that are stored in separate text files (e.g., a data frame with age, race, study site, and head motion). Note, ABCD data are available in multiple formats, including raw data files as well as community-processed output (Feczko et al., 2021), but the data output discussed in the present paper refers to the curated output.

### 3.5 Organization and structure of data files

The structure and organization of ABCD data is consistent across variable types. Tabulated data from the NIMH Data Archive are downloaded as.txt or.Rds files, each of which contains a different family of variables. Using one variable for example, cbcl_scr_syn_external_t is the t-score of youth externalizing behaviors as assessed by the parent-reported Child Behavior Checklist (CBCL). This variable is housed in a data file—available as a.txt or.Rds file—called *abcd_cbcls01*, along with many other CBCL subscale variables. When identifying your variables of interest, it is important to know both specific variable names (e.g., cbcl_scr_syn_external_t) and the text files in which those variables are stored (e.g., *abcd_cbcls01*). This information can be found on the NDA ABCD data dictionary or the DEAP portal by searching keywords (e.g., child behavior). For example, on the data dictionary, you will first find the name of the measure of interest (e.g., ABCD Parent Child Behavior Checklist Scores Aseba (CBCL)) and the short name for this measure (e.g., abcd_cbcls01). The short name will be of most relevance here as it corresponds to the measure name in the curated data release.txt or.Rds file. In the data dictionary, you will find additional information such as a description for each variable, the data type (e.g., integer, string), and any additional notes (e.g., how the variable was scored). It is important to note that for some variables, such as the CBCL, the raw and tabulated scores are listed as two separate measures so it is worth double checking that you are using the correct version of your measure of interest.

### 3.6 Availability of the data

Just like any longitudinal study, not all ABCD data are measured at every wave of data collection. As such, it is important to identify whether your variables of interest have been assessed at the waves in which you propose to study them. The ABCD Study has excellent documentation of assessment protocols for every wave (see https://abcdstudy.org/scientists/protocols/). At a high level, data collection protocols outline the following: an annual comprehensive battery of physical health, mental health, substance use, culture and environment, and biospecimens; a bi-annual (every 24 months) MRI scan; and intermediate mid-year phone assessments of youth behavior, substance use, and affect. In addition to determining which measures are available at each wave of data collection, it is also important to consider missing data. When possible, it is important to consider whether data are missing at random or missing not at random — for instance, if participants with missing MRI data are more likely to have a clinical diagnosis — as this will have important implications for our statistical models (Matta et al., 2018, Schafer and Graham, 2002).

### 3.7 Covarying with caution

When working with a dataset like ABCD, with tens of thousands of variables, it can be tempting for researchers to include a host of covariates in their models. However, this approach should be avoided as it can lead to model overfitting, as well as increased risk of Type 1 error. Often, covariates, especially socioeconomic and demographic factors, are included because of norms within the research environment. Rather, the inclusion of covariates should be linked directly to hypotheses and supported by a clear theoretical justification for each covariate (Wysocki et al., 2020). In other words, we should pay as much attention to our covariates as we would to our independent and dependent variables at every stage of the study life cycle, from analysis plan to interpretation of our results (see *Analyses Plan* for further discussion). Further, it is important to consider the impact of including certain variables, such as “race/ethnicity”, as a covariate in our models in terms of the subsequent inferences that can be drawn (see *Justice, Equity, Diversity, and Inclusion* for further discussion). Even with specific hypotheses, researchers should consider the meaning of each dataset variable and what it can actually measure. In other words, including covariates like “race/ethnicity” does not simply “control” for the effects such factors may have on a child’s development and the nuance of their unique experience (e.g., such variables do not account for experiences of racism). Therefore, it is important to acknowledge the complexity of the construct we are trying to measure and what can be captured by an operationalized variable in quantitative analyses. This is particularly important to keep in mind when using a secondary data set in which we are limited to the kind of variables collected. We also recommend consulting any DCN ABCD special issues and other protocol papers (e.g., Hagler et al., 2019) when deciding which covariates to include.

### 3.8 Details of the data

Once it is clear at what time points the variables of potential interest have been measured, a deeper dive is needed to determine whether these measures/variables are appropriate for your study. Some guiding questions include:

**Table:** 

• What does the measure assess? Is the measure assessing the same construct you would like to model? • Who is reporting (parent, child, teacher, American Community Survey/census, etc.)? • What are the descriptive statistics (e.g., mean, variance/standard deviation, sample size) of the variable? Is the distribution of the variable skewed or normal? Does this matter for your question? • Have you excluded participants based on recommendations by the ABCD Data Analytics and Informatics Core (DAIC)? See below for further details on DAIC. • Has the measure been used in previous studies (e.g., prior to ABCD)? • Is the measure reliable? Is it valid? Is it invariant across time? • How have the responses to the measure been coded? Will you need to recode any variables for your uses? • Example: An assessment of youth perceptions of neighborhood safety (e.g., “My neighborhood is safe from crime”) is coded using a Likert-style response, with 1 = Strongly disagree and 5 = Strongly agree. If you aim to calculate a summed score or factor with higher scores indicating greater feelings of danger, you may want to reverse code these. • If the measure includes more than one indicator, will you use a summed or averaged score, or will you create a factor?

Several of these questions are easy to answer, even before downloading any data. For instance, in order to view the exact wording, reporter, coding of responses, and descriptive statistics of a variable, you may log on to DEAP, click on “Explore,” and type into the search bar the variable name (e.g., cbcl_scr_syn_external_t), and the necessary information will pop up. Float your mouse over the variable, and you’ll see its descriptive statistics. However, several other questions—such as those pertaining to transformations, factor analysis, reliability and validity—require more thought, effort, and specificity according to the aims of your study, meditations on which are generally beyond the scope of this paper. The ABCD Data Analytics and Informatics Core (DAIC) coordinates harmonized MRI acquisition across sites, establishes standardized image processing and extraction, ensures quality control of imaging data, and engineers tools for data sharing and statistical analysis. Full inclusion and exclusion criteria may be found in the ABCD 4.0 release notes. Some additional helpful resources as you work through the questions outlined above include: Goretzko et al. (2021); McNeish and Wolf (2020); van de Schoot et al. (2012).

### 3.9 Preparing your data for analysis

Once you have selected your variables of interest, you must now decide how to quality control and prepare your data in advance of running your statistical models. As with all of your work with ABCD data (and any data for that matter), we strongly recommend adopting a transparent workflow for your quality control process and, when possible, preregistering planned quality control steps in advance. Clearly tracing each step will increase transparency and enable the reproducibility of steps from raw data to quality-controlled data that are ready for use in your statistical models. We recommend that you document any deviations from your pre-registered analysis plan, and include information about these changes in any resulting manuscripts. This can in part be done on OSF, which as of December 2021 allows users to update their registrations and transparently reflect changes to the initial registration. This feature includes an interface that allows readers to easily view changes made to the initial registration (for details, visit https://help.osf.io/hc/en-us/sections/4414482864279-Updating-Registrations). Further, we strongly encourage you to make your quality control scripts publicly available alongside your manuscript so that other ABCD users can see exactly how the data were processed.

Before you start quality controlling your data, we also encourage you to consult existing literature that uses ABCD data to see if there are any published guidelines or protocols for your variables of interest. For example, guidelines for working with the imaging (e.g., Chaarani et al., 2021; Hagler et al., 2019), cognition (Luciana et al., 2018), puberty measures (Cheng et al., 2021, Herting et al., 2021), assessment of culture and environment (Gonzalez et al., 2021) and substance use behavior (Lisdahl et al., 2018) have been published to date. If you are interested in utilizing the neuroimaging data, readers must first consult release notes and protocol papers for explicit directions on what inclusion/exclusion criteria needs to be applied to some modalities. For example, Hagler et al. (2019) recommends specific exclusion criteria for resting state (e.g., number of frames) and structural MRI (e.g., quality control scores) that readers can refer to and utilize. Similarly, Cheng et al. (2021) recommend using standardized, publicly available, processing procedures for the salivary hormone pubertal data, such as those proposed by Herting, Uban et al. (2021) (Scripts available here: https://figshare.com/articles/software/R_scripts/12673754).

If a new release is issued during the course of your project, it would be worthwhile to closely check the Release Notes, as they will contain details about any errors that may have been found in previous releases. Further, variable names can change across releases for a variety of reasons (e.g., refined quality control variables for the imaging data), which will be noted in the Release Notes. We recommend using the most recent data release in all projects, taking into consideration any upcoming releases in your project timelines, and specifying the release number in any related manuscripts. This underscores again the need for having scripts to reproduce your analysis if an error is found in a previous version of the released data. There are, however, other types of errors that occur in the acquisition of the data that may be long standing and are not fixed between data releases. As a result, it is important to look at the extant ABCD studies that have used your variables of interest. For the functional imaging data, for example, papers highlight issues in scanner software harmonization (Nielson et al., 2018), fMRI task design (Bissett et al., 2021), and reliability of task data (Kennedy et al., 2021). However, given the breadth of data in ABCD, it is likely that you may need to quality control your data in a specific way to suit the needs of your research question(s). Software like RMarkdown, which allows you to weave together narrative text and code, can be very useful in making your workflow reproducible because you can document each decision made in the quality control process alongside the associated code. The inclusion of flowcharts is also recommended so that you can illustrate changes, such as your sample size, at each quality control stage. RMarkdown provides useful functions, such as “DiagrammeR”, to create such flowcharts. Although a comprehensive list of quality control actions you may want to consider is beyond the scope of the current paper, some primary considerations include:

**Table:** 

• Have you reduced your data to the data collection wave(s) of interest? • Have you checked that your variables are in the correct format (e.g., numeric, categorical, etc.)? • Have you checked the distribution (via histograms, QQ-plots) of your data, and does it meet the assumptions of your planned statistical tests (e.g., normal distribution of residuals for linear regression)? • If your data are skewed, are you going to transform the data? • How are you going to treat extreme values or outliers (see section on Outliers for further consideration of this point)?

## 4 Planning your analysis

In any quantitative study, deciding on an analysis plan can be a daunting task, especially in the case of an expansive secondary dataset like ABCD. Issues in working with secondary data may be further muddied for those who are working on a preregistration or registered report and are trying to refrain from looking at the data as much as possible. Additionally, thoroughly understanding the structure of the dataset and the expectations from extant literature is an essential part of planning your analyses. In this section, we discuss how your pre-registered plan can include analyses of reliability and sensitivity of your model and expectations regarding the distribution, magnitude, and meaning of these effect sizes. We have also included a *Plan Ahead Checklist* for readers to consider when devising their analysis plan:

**Plan Ahead Checklist**

□ Have you familiarized yourself with existing research that has used the ABCD Study data?
□ Do you know the accessibility, type, timing, and structure of your study variables?
□ What are your covariates and why?
□ Have you detailed all your QC steps?
□ What do you need to know about the data to create an analysis pipeline?
□ Do you need to subsample the data? If so, what method will you use?
□ Have you pre-registered your analysis plan?

### 4.1 Subsetting the data

We recommend taking advantage of the large sample size to work with subsamples of data, a strategy that will help to gain familiarity with the data structure without unveiling the exact data that will be used in one’s analysis. Subsamples are extremely useful for testing pipelines or generating hypotheses, while ensuring that some data are kept hidden. Working with a subsample may even be necessary to provide the level of detail required for a pre-registration or registered report, especially when it comes to processing variables and quality control protocols.

When working with a subset of the data, it may be useful to consider strategies such as stratified sampling and population weighting. These strategies will help to create a subsample that still reflects the population as a whole. Additionally, in a study as large as ABCD, splitting the dataset into subsamples matched on relevant variables (e.g., age, gender, etc.) can be used to conduct within-study replications (Meredith et al., 2022, Tomasi and Volkow, 2021). As there are many ways to subsample data, we recommend looking at previously published papers using ABCD data to identify the method that is best suited for your question.

### 4.2 Considering effect sizes

Larger datasets have the great advantage of providing insight into smaller effects that were previously undetectable (Owens et al., 2021, Paulus et al., 2019). However, with this increased power comes increased responsibility for the researchers to identify meaningful and relevant effect size (Dick et al., 2021, Funder and Ozer, 2019). Prior to your analyses, meta-analyses may be useful in establishing expected effect sizes or effect size ranges for the variable of interest (or related variables), enabling authors to pre-register expectations and approaches to interpretations of effect sizes. Although an exact answer may not be available in the extant literature, there is likely guidance on what effect size would you expect based on other developmental cohorts, similar constructs, or established parallel metrics of risk markers (Damme et al., 2021) or public health benefit (Vargas et al., 2020) in the field. Another critical consideration in developmental cohorts is whether the effect is expected to accumulate over time. In such cases a small effect may have a large influence over time. It may also be important to estimate the reliability of effect sizes when they are expected to be small by using hold-out samples or sensitivity analyses (Saltelli, 2004).

In reporting data, it may also be important to reframe the effect sizes into the most clinically useful metric possible. For example, if you are exploring a potential risk marker for psychopathology, you may want to provide the relative risk ratio or sensitivity of that measure rather than chi-square alone. In other cases, it may be useful to provide the percentage of variance explained by a variable (R²) or the difference attributed to a variable (Cohen’s d). Planning ahead will allow you to identify clear and reasonable benchmarks for relevant effect sizes and help you report the type of effect size that provides the most insight into the variable of interest.

### 4.3 Working with outliers

It is critical to plan for extreme values, or outliers, in your data. Extreme values do not always reflect errors in a dataset. Instead, they may reflect several categories of outlying data points that include error outliers, valid/interesting outliers, and influential outliers (Aguinis et al., 2013). Researchers most often are concerned with identifying outliers that reflect errors (error outliers) or influence statistical models (influential outliers) and seek to minimize their effects (Aguinis et al., 2013, Leys et al., 2019). However, many statistical approaches to outliers risk reducing the extreme values of interest (valid/interesting outliers), which may impact your conclusions (Tong, 2019). Planning ahead for the detection of error outliers, defining data of interest, and examining the model influence of extreme values can help preserve the extreme values that are of interest to your research question.

#### 4.3.1 Interest outliers

In large datasets, eliminating extreme values should be done with care, as extreme values may also reflect data of interest. Indeed, the appeal of larger studies such as the ABCD Study is that samples include individuals with both high and low values on outcomes of interest (Bjork et al., 2017, Volkow et al., 2018). As a result, simplistic methods that remove all extreme values could potentially eliminate 5% of the most theoretically interesting data (Osborne and Overbay, 2004, Tong, 2019). These extreme values may be especially critical when the model is intended to examine a normal to abnormal spectrum or is aimed at understanding atypical behaviors. In such cases, it may be expected that the tail ends of the distribution would have the greatest influence on the model.

#### 4.3.2 Error outliers

There is always the possibility of sincere error in the data sets. It is therefore critical, when possible, to check for sincere errors in the data. (Note: If you suspect that you have detected sincere errors in the ABCD Study dataset, then this should be reported back to the ABCD Coordinating Center at abcd-cc@ucsd.edu). For these checks, it is important to look for only clear errors (i.e., impossible data). To avoid unnecessarily reducing data, be cautious and conservative in identifying error outliers, only considering exclusion if data clearly indicate that there was an error in administering, reporting, or transcribing the data. After data have passed some basic checks for feasibility and error, the resulting sample can be examined for other outlier influences on the distribution of the valid data to detect additional error or other forms of outliers. However, it should be noted that the experts from the ABCD working groups quality control the data and check for impossible data points prior to each release, which should reduce the possibility of error outliers.

#### 4.3.3 Influential outliers

Influential outliers are extreme values that are accurate and influence your model but are not relevant or interesting to the theoretical question at hand. There are many possible analytic strategies to address such outliers, such as exclusion, regression, structural equation modeling, and multilevel modeling (Aguinis et al., 2013). It is important to plan ahead for the types of approaches that are best suited for the current research question. One should consider the power to model the effects of these influential outliers and their contribution to future research. If there are additional insights to be gained from modeling these variables that may be useful to other studies, then consider adding appropriate parameter information (e.g., effect sizes of model influence) to emphasize the influence of the outliers for future research.

#### 4.3.4 Outlier detection methods

In large datasets, such as the ABCD Study, rule-of-thumb (e.g., 3 standard deviations from the sample mean) may not be appropriate or ideal as it may exclude hundreds of valid data points. Other statistical outlier tests, e.g., Grubb’s or Dixon’s test, that test one value at a time and iteratively trim the dataset may be impractical for large datasets (Walfish, 2006). Instead, it may be important to consider multivariate approaches to addressing outlier data that are informed by your current research question and expectations of the data (Finch, 2012, Selst and Jolicoeur, 1994, Tong, 2019). Finally, other highly recommended steps, including reliability and sensitivity analyses (Saltelli et al., 2008), may also aid in reducing concerns regarding the influence of particular influential outliers. Here, we have included a non-exhaustive list of considerations when creating an analysis plan**:**

**Table:** 

• Are you already familiar with the structure/details of your variable(s) of interest? • What do you need to know about your variables in order to create an analysis plan? • Are the variables present at all waves? • What format are the variables in? • What cleaning procedures might you need to do? • Do you need to split/subsample the data? • Consider population weighting, stratified sampling, matched samples • Effect sizes: • What is expected based on similar variables? • What would be a meaningful effect? • What effect size would be most useful for the translation of this research? • Outliers: • Is there an error in the data? What would impossible data look like? • How will you detect influence outliers? Could you reasonably model the influence of these outliers? • What are the relevant features of your data to examine outliers? • What sort of data distribution do you expect based on your research questions?

## 5 Justice, equity, diversity, and inclusion

Careful consideration of justice, equity, diversity, and inclusion is integral to any research study. In this section, we outline major themes, questions, and discussion points related to these efforts as they relate to the ABCD Study and other large-scale studies in the population. However, this section is not intended to serve as an exhaustive manual of directions but rather as a conversation and starting point for researchers to keep in mind, revisit, and carefully discuss at every stage of a research project.

### 5.1 The use of demographic variables in secondary data analysis

One important consideration is the use of demographic variables in secondary data analyses of the ABCD Study. Purposefully reporting and analyzing sample demographics is crucial for reproducibility, generalizability, and development of appropriate interventions. However, careful thought should be given as to whether and how such variables should be included in analyses. For example, including racial/ethnic variables as covariates should not be a “default” analysis approach (Wysocki et al., 2020). In addition, due to variability in use of labels and operationalizations of terms which can cause confusion in the literature, clear definitions used by the researchers should always be included and explained within the manuscript (e.g., “Although these terms are not interchangeable, we will use “Hispanic” as a shortened version of Hispanic/Latinx to indicate a population of people who are either from or descended from Spanish and Portuguese speaking Latin American countries. For more details, please see our methods section”; (Montoya-Williams et al., 2021), p. 2). This can be challenging as such decisions often involve a balance of highlighting terms preferred by the study population (e.g., only 3% of Hispanics use the term ‘Latinx’), maintaining fidelity to the terms the participants were originally presented with while participating in the study, and using inclusive, current language (e.g., gender-inclusive terms such as ‘Latinx’ or ‘Latine’; Pew Research Center, 2020b). In certain instances, the researchers could choose *not* to report analyses split by demographics, to minimize potential for damaging interpretations and avoid recapitulation of harmful narratives.

### 5.2 A closer look at race and ethnicity

The importance of how we measure race and ethnicity also extends to our coding of these variables in our sample, as this can fundamentally alter models and interpretations of results. A commonly used coding schema for race and ethnicity in the ABCD Study, variable “*race_ethnicity”* derived from the NIH Minimum Reporting guidelines and the Office of Management and Budget (OMB) standards used by the US Census Bureau, collapses these constructs into a five-level combined ethnoracial construct (categories: Hispanic, non-Hispanic White, non-Hispanic Black, non-Hispanic Asian, and other). Although this construct may be useful at encapsulating differences between white and Black and Hispanic populations, researchers should be mindful that these categories are subject to political and historical context, as demonstrated by the changes in these labels over time (Pew Research Center, 2020a), and may contribute to the ongoing erasure, invisibility, and lack of recognition of various important populations, such as those that identify as American Indian and Alaska Native, Native Hawaiian or Other Pacific Islander, Middle Eastern or North African, or individuals that identify as multiracial.

### 5.3 Operationalization of demographics within and beyond the ABCD study

Although no coding schema is perfect at encapsulating the rich and diverse identities of our research populations, researchers should be mindful and explicit of their selected operationalization of race and ethnicity. When testing group differences by gender, race, ethnicity, SES, and their intersections, researchers should consider including measures developed from the vantage point of the identified population, including measures that characterize larger systems of inequity. For example, measures such as the Mexican American Cultural Values Scales Modified for youth (Knight et al., 2010); Vancouver Index of Acculturation and Multi-Group Ethnic Identity (Ryder et al., 2000); Perceived Discrimination Scale (Williams et al., 1997); Life Events Scale (Hoffman et al., 2019a, Hoffman et al., 2019b, Tiet et al., 1998); Neighborhood Safety/Crime Survey (from the PhenX Toolkit and adapted from Echeverria et al., 2004 and Mujahid et al., 2007); and the School Risk and Protective Factors Survey (derived from the School Social Environment Section in the PhenX Toolkit and adapted from Arthur et al., 2002 and Harter, 2006) are all available in the ABCD dataset and can be incorporated to better identify why any observed differences exist and ascertain the structural systems that perpetuate them (see Gonzalez et al., 2021 and Zucker et al., 2018 for more detail on the culture and environment assessments that were selected for inclusion in the ABCD protocol). In addition, if researchers do decide to analyze variables related to race and ethnicity of the sample, it is recommended that they include a thoughtful discussion and interpretation of the findings, to avoid possible negative interpretations of the findings by the readers. For example, upon identifying a relation between SES and a brain metric–or when studying executive functioning among minoritized youth– culturally- and contextually-informed alternative explanations to the deficit framework should be considered (Miller-Cotto et al., 2021, Nketia et al., 2021).

### 5.4 An intersectional lens

Intersectionality, a multi-axis framework originally coined by Kimberlé Crenshaw (Crenshaw, 1989), argues that the intersection of an individual’s social identities (e.g., race *and* gender, together) must be taken into account to fully capture an individual’s lived experiences — as opposed to studied in isolation. We encourage researchers to consider social identity metrics including–but not limited to–race, ethnicity, gender, sexuality, and class, holistically. When appropriate, researchers may wish to adopt an intersectional lens for their statistical approaches.

For example, in a recent study, Hong et al. (2021) applied an unsupervised, cross-modal integration and clustering approach (called ‘Similarity Network Fusion’) and were able to decompose associations between environmental disadvantages and brain development in a large sample of school-aged children from the ABCD Study. One advantage of this procedure is high reproducibility of the observed subtypes which can enhance prediction of individual differences in mental health symptoms. Other statistical approaches that should be considered are canonical correlations or partial least squares analysis to potentially account for collinearity among measures (Wold et al., 1984) as well as dimensionality reduction techniques for, and/or factor analyses of, sociodemographic variables to identify meaningful subclusters that may better represent the sample (Wang and Zhang, 2017, Xiao et al., 2018).

Researchers should intentionally question the importance of including social identity variables in their analysis and discuss the ideal operationalization of these constructs. Guiding questions could include: “How do we measure and conceptualize race and ethnicity and how do these constructs overlap?” Additionally, “how do race and ethnicity differ and how are they unique?”; “Why are these variables important for our research question and how could we implement them into our conceptual model?” (Martinez et al., 2021a, Martinez et al., 2021b). Additionally, the messages that inclusion of variables in analyses may communicate back to communities should be given careful consideration. Researchers may wish to include an explanation of their decision regarding inclusion of variables in analyses.

In relation to intersectionality, researchers may find themselves asking “what else could be going on here?” and “what are the limitations of what we have explored thus far?”. It is worth noting that inclusion of two variables, for example as interaction terms, may not fully capture the extent of people’s lived experiences. Researchers should feel empowered to include additional variables in analyses when appropriately justified. While the ABCD Study offers an important opportunity to explore questions related to intersectionality, smaller scale studies with more focused research questions may be better suited to address questions–perhaps arising from the ABCD Study–that relate to social identity and incorporate an intersectional approach.

### 5.5 Empowering research participants to be active agents in community-based research

When considering who is included in the group being studied, we encourage engaging the communities that are impacted by the research in the processes that determine how research is done (see also Rosenthal, 2016). With a substantial focus on environmental pathways affecting neurobiology and its development in the ABCD Study, it is no longer enough to simply collect data from participants in the community without also considering how the community might benefit from this research. The ABCD Study has engaged youth, families, and educators from the beginning of study development to ensure that the needs of participants involved in the study are met. For example, the ABCD Outreach and Dissemination workgroup developed multi-pronged engagement strategies to help families understand the potential impact of the study and opened dialogues with families to learn about their concerns and inform ongoing study design (Hoffman et al., 2018). We suggest fostering a partnership between the research team and the communities who reflect the researched sample to ensure that researchers gain an understanding of the social context in which community members assess the risks and benefits of research (see Whitmore and Mills, 2021 for a discussion of co-produced developmental science). This is particularly important at sites with a large percentage of individuals from historically excluded communities, where cultural and historical context are highly relevant. One suggestion as we move forward is the formation of Community Advisory Boards (CABs), often used in health disparities research, which formalize the researcher-community partnership by centering the concerns and priorities of the community in the researchers’ agenda. Criteria and guidelines in formulating CABs have been well-established (Newman et al., 2011). CABs can be comprised of community representatives (from study participants or nonmembers) who formally help inform research protocols, provide researchers with real life examples of issues under study, voice the concerns of the community, assist in developing community education resources, and help disseminate research findings. Incorporating CABs into research can provide a framework for developmental neuroscientists to create and sustain authentic community–academic partnerships. Such efforts are underway and the ABCD Consortium is planning to establish community liaison boards (which include local leaders, educators, and families) (Hoffman et al., 2018).

We acknowledge that it is not always possible for researchers working with secondary datasets like ABCD to engage with participants and study communities directly. Nonetheless, we strongly encourage researchers to make every effort to disseminate their research findings to relevant communities (e.g., youth, school, parent, or patient groups) through science communication activities alongside the publication of peer-reviewed journal articles. Doing so will ensure that both the scientific community and the public are made aware of advancements in our understanding of adolescent development and behavior as they emerge.

### 5.6 Additional resources for considerations related to justice, equity, diversity and inclusion

**Table:** 

Foundational work • American Psychological Association, APA Task Force on Race and Ethnicity Guidelines in Psychology. (2019). Race and Ethnicity Guidelines in Psychology: Promoting Responsiveness and Equity. • Alvidrez, J., Castille, D., Laude-Sharp, M., Rosario, A., & Tabor, D. (2019). The National Institute on Minority Health and Health Disparities Research Framework. *American Journal of Public Health*, *109*(S1). https://doi.org/10.2105/ajph.2018.304883 • Palmer, R. C., Ismond, D., Rodriquez, E. J., & Kaufman, J. S. (2019). Social Determinants of Health: Future Directions for Health Disparities Research. *American journal of public health*, *109*(S1), S70–S71. https://doi.org/10.2105/AJPH.2019.304964 • Public Health Critical Race Praxis • Ford C.L., Airhihenbuwa C.O., Critical race theory, race equity, and public health: toward antiracism praxis. Am. J. Public Health. 2010a S30-S35 • Ford C.L., Airhihenbuwa C.O., The public health critical race methodology: praxis for antiracism research. Soc. Sci. Med.1390-13982010b • Ford C.L., Airhihenbuwa C.O.. Commentary: just what is critical race theory and what’s it doing in a progressive field like public health? Ethn. Dis. 2018 223-230 • Simmons, C., Conley, M. I., Gee, D. G., Baskin-Sommers, A., Barch, D. M., Hoffman, E. A., Huber, R. S., Iacono, W. G., Nagel, B. J., Palmer, C. E., Sheth, C. S., Sowell, E. R., Thompson, W. K., & Casey, B. J. (2021). Responsible Use of Open-Access Developmental Data: The Adolescent Brain Cognitive Development (ABCD) Study. *Psychological science*, *32*(6), 866–870. https://doi.org/10.1177/09567976211003564 Intersectional frameworks • Crenshaw, K. (1989). "Demarginalizing the Intersection of Race and Sex: A Black Feminist Critique of Antidiscrimination Doctrine, Feminist Theory and Antiracist Politics," *University of Chicago Legal Forum: Vol. 1989, 1*(8). http://chicagounbound.uchicago.edu/uclf/vol1989/iss1/8 • Sidanius, J., Hudson, S.T., Davis, G., & Bergh, R. (2018). The Theory of Gendered Prejudice: A Social Dominance and Intersectionalist Perspective. *The Oxford Handbook of Behavioral Political Science.* DOI: 10.1093/oxfordhb/9780190634131.013.11 Collection and coding of demographic variables • Bureau, U.S.C. (2021, October 8). *2010 census race and Hispanic Origin Alternative Questionnaire Experiment*. Analyses and interpretation • Vyas, D. A., Eisenstein, L. G., & Jones, D. S. (2020). Hidden in Plain Sight - Reconsidering the Use of Race Correction in Clinical Algorithms. *The New England journal of medicine*, *383*(9), 874–882. https://doi.org/10.1056/NEJMms2004740 • Smith, N. (2020, September 9). *Beyond the boxes, part 1: Guiding questions for thoughtfully measuring and interpreting race in Population Health Research*. IAPHS. https://iaphs.org/beyond-the-boxes-part-1-guiding-questions-for-thoughtfully-measuring-and-interpreting-race-in-population-health-research/ Racism • Brondolo, E., Ng, W., Pierre, K.-L. J., & Lane, R. (2016). Racism and mental health: Examining the link between racism and depression from a social cognitive perspective. In A. N. Alvarez, C. T. H. Liang, & H. A. Neville (Eds.), The cost of racism for people of color: Contextualizing experiences of discrimination (pp. 109–132). American Psychological Association. https://doi.org/10.1037/14852-006 • Ford, C. L., Griffith, D. M., Bruce, M. A., & Gilbert, K. L. (2019). *Racism: Science & tools for the Public Health Professional*. American Public Health Association. • Alvarez, A. N., Liang, C. T. H., & Neville, H. A. (Eds.). (2016). The cost of racism for people of color: Contextualizing experiences of discrimination. American Psychological Association. https://doi.org/10.1037/14852-000

## 6 Conclusion

This paper is meant to serve as a reference and guide for researchers conducting or reviewing studies using ABCD data, but many points can similarly be applied to other multi-method, longitudinal, and developmental datasets. This guide is not meant to be prescriptive, and some content may vary in relevance depending on the research question being addressed. Nevertheless, our hope is that this guiding framework equips readers with a better sense of how to navigate large-scale projects such as the ABCD Study when conducting or reviewing secondary data analyses.

## CRediT authorship contribution statement

**Carlos Cardenas-Iniguez:** Conceptualization, Writing – original draft, Writing – review & editing. **Natasha Chaku:** Conceptualization, Writing – original draft, Writing – review & editing. **Katherine SF Damme:** Conceptualization, Writing – original draft, Writing – review & editing. **Ece Demir-Lira:** Conceptualization, Writing – original draft, Writing – review & editing. **Brandee Feola:** Conceptualization, Writing – original draft, Writing - Review & Editing;Landry Goodgame Huffman: Conceptualization, Writing - Original Draft, Writing – review & editing. **Victoria Guazzelli Williamson:** Conceptualization, Writing - Original Draft, Writing - Review & Editing; Niamh MacSweeney: Conceptualization, Writing – original draft, Writing – review and editing. **Elizabeth A. McNeilly:** Conceptualization, Writing – original draft, Writing – review & editing. **Kalina J Michalska:** Conceptualization, Writing – original draft, Writing – review & editing. **Kate Mills:** Conceptualization, Writing – review & editing, Supervision. **Divyangana Rakesh:** Writing – original draft, Writing – review & editing. **Natalie Saragosa-Harris:** Conceptualization, Writing – original draft, Writing – review & editing. **Maximilian Scheuplein:** Conceptualization, Writing – original draft, Writing – review & editing. **Lucy Whitmore:** Conceptualization, Writing – original draft, Writing – review & editing.

## Declaration of Competing Interest

The authors declare that they have no known competing financial interests or personal relationships that could have appeared to influence the work reported in this paper.

## Data Availability

No data were used for the research described in the article.
